# A Single Catalytic Endolysin Domain Plychap001: Characterization and Application to Control *Vibrio parahaemolyticus* and Its Biofilm Directly

**DOI:** 10.3390/foods11111578

**Published:** 2022-05-27

**Authors:** Luokai Wang, Xiaochen Ju, Yu Cong, Hong Lin, Jingxue Wang

**Affiliations:** Food Safety Laboratory, College of Food Science and Engineering, Ocean University of China, No. 5, Yushan Road, Qingdao 266003, China; luokaiwang@outlook.com (L.W.); 18562760993@163.com (X.J.); ouccongll@163.com (Y.C.); linhong@ouc.edu.cn (H.L.)

**Keywords:** endolysin, *Vibrio parahaemolyticus*, single catalytic domain, biofilm, food safety

## Abstract

Endolysins are enzymes used by bacteriophages to cleave the host cell wall in the final stages of the lytic cycle. As such, they are considered promising antibacterial agents for controlling and combating multidrug-resistant (MDR) bacteria. However, the application of endolysins targeting Gram-negative bacteria is greatly hindered by the outer membrane on these bacteria. Lysqdvp001, an endolysin with modular structure, has been reported as one of the most efficient endolysins against the Gram-negative bacterium *Vibrio parahaemolyticus*. In this study, Plychap001, the truncated recombinant catalytic domain of Lysqdvp001, was demonstrated to exhibit a direct and efficient bactericidal activity against broad spectrum of *V. parahaemolyticus* strains. Plychap001 was shown to be highly stable and retain high bactericidal activity at high temperatures, over a wide pH range, and at high NaCl concentrations. Plychap001 also exhibited a synergistic lytic effect with EDTA. Additionally, Plychap001 was found to efficiently degrade and eliminate *V. parahaemolyticus* biofilms on polystyrene surfaces. Our study establishes Plychap001 as a promising method for controlling *V. parahaemolyticus* in the food industry.

## 1. Introduction

*Vibrio parahaemolyticus*, a Gram-negative bacterium commonly found in seawater, is one of the major causes of seafood-associated infections worldwide [1,2,3]. Consumption of food contaminated by *V. parahaemolyticus* may lead to gastrointestinal illness with symptoms such as nausea, vomiting, abdominal pain, diarrhea, and acute gastroenteritis [4]. During the last few decades, excessive use of antibiotics in medical treatment and aquaculture has resulted in the emergence of *V. parahaemolyticus* strains resistant to single or multiple antibiotics [5,6]. Adaptive strategies such as uptake of multi-drug resistance plasmids or genes and biofilm formation enable *V. parahaemolyticus* to withstand antibiotics or reduce their efficiency [7,8], which makes this bacterial species a growing threat to public health. Alternative methods to combat multi-drug resistant (MDR) *V. parahaemolyticus* are therefore urgently needed.

Endolysins are produced by phages during the last stages of the lytic cycle to cleave peptidoglycan (PG) bonds within the host cell wall, which not only allows the release of phage progeny but also results in death of the host bacterial cells [9,10,11]. Endolysins have also been found to be highly effective against bacteria in biofilm and have been demonstrated to significantly reduce biofilm formation as well as induce its degradation [12,13]. Importantly, bacteria are less likely to develop resistance to endolysins than to conventional antibiotics due to the long-term co-evolution of phages and host bacteria [14]. Up to now, many endolysins targeting different Gram-positive bacteria have been developed for application [15,16,17,18]. In contrast, the application of endolysins against Gram-negative bacteria is still limited due to the presence of an outer membrane that obstructs direct access to PG [19,20,21]. Nevertheless, relevant studies have demonstrated that some endolysins are capable of breaking through the outer membrane barrier effect of Gram-negative bacteria with the help of outer-membrane permeabilizers (OMPs) [21], cationic peptides [22], or antimicrobial peptides [23]. An attractive strategy is the use of endolysins or endolysin-antimicrobial peptide fusion proteins that can eliminate the protective effect of the outer membrane and lyse bacteria without pretreatment with other OMPs. For instance, Nie et al. generated antimicrobial peptide-endolysin chimeric proteins that were able to break through the outer membrane of *Salmonella* and kill these Gram-negative bacteria with high specificity and efficiency [23]. In addition, Guo et al. identified and characterized endolysin LysPA26 that has strong bactericidal activity against MDR *Pseudomonas aeruginosa* without pretreatment with OMPs [24].

In a previous study, we identified Lysqdvp001 as a Gram-negative bacteria-targeting endolysin with modular structure but found that full-length Lysqdvp001 cannot penetrate the outer membrane of *V. parahaemolyticus* without the aid of OMPs [21]. Modular structure is a common feature of Gram-positive endolysins and their catalytic domains have been reported to also achieve cell lysis independently [15,18]. Here, we demonstrate potent lytic activity of the single catalytic domain Plychap001 originating from the endolysin Lysqdvp001 against *V. parahaemolyticus* cells in vitro and in mature biofilm. The findings of this study show that Plychap001 maintains high stability under different reaction conditions, that it is able to efficiently degrade mature biofilms, and that it possesses a broad antibacterial spectrum against multiple strains of *V. parahaemolyticus*. Therefore, this study establishes Plychap001 as a promising antibacterial agent for controlling MDR *V. parahaemolyticus* in the food industry.

## 2. Materials and Methods

### 2.1. Bacterial Strains

*V. parahaemolyticus* 17802, *Salmonella typhimurium* 14028 and *Staphylococcus aureus* 43300 were purchased from the American Type Culture Collection (ATCC). *V. parahaemolyticus* isolates VP 1.1, VP 1.3, VP 2.2, VP 3.1, VP 3.2, VP 4.1, VP 304, VP 461, VP 800 and VP l4-90, *Vibrio harveyi* V1, *Shewanella baltica* S1, *Acinetobacter baumannii* A1, *Pseudomonas fluorescens* P1, *Aeromonas hydrophila* A1, *Pseudomonas putrefaciens* P1, *Vibrio anguillarum* V1 were stored in the Food Safety Lab at the Ocean University of China. *E. coli* DH5α and *E. coli* BL21 (DE3) were purchased from Sangon Biotech (Shanghai, China). All marine bacterial species were routinely cultured in the Zobell 2216 marine medium (Hopebio, Qingdao, China), whereas the other bacteria were grown in LB medium (Hopebio, Qingdao, China). Working concentrations of antibiotics were added into the growth medium when necessary.

### 2.2. Cloning, Expression and Purification of Plychap001

PCR primers (chap-F:5′-CGTCA TATGC ATCAT CATCA CCATC ACGAC-3′ and chap-R:5′-TCGAA GCTTT TATTG ATGCG GATTA CGATA G-3′) were designed based on the optimized gene sequence of Plychap001 to amplify the target PCR fragment (327 bp) using 2 × Phanta^®^ Flash Master Mix (Vazyme, Nanjing, China). After double digestion with NdeI (NEB) and HindIII (NEB), gene amplification products were ligated with a similarly digested pET-30a vector to generate the recombinant plasmid pET-30a-Plychap001. Successful insertion was confirmed by Sanger sequencing at Sangon Biotech (Shanghai, China). The recombinant pET-30a-Plychap001 was then transformed into *E. coli* BL21 (DE3) cells. After overnight culture, 10 mL of the expression strain was diluted at 1:100 ratio in 1 L LB media containing 50 μg/mL kanamycin, followed by incubation at 37 °C and 180 rpm for 2–3 h until OD_600 nm_ reached 0.6. The expression of Plychap001 was induced with isopropyl β-D-thiogalactopyranoside (500 μM) at 16 °C for 7 h. Bacteria were subsequently collected by centrifugation at 3000× *g* and 4 °C for 15 min. The pelleted cells were resuspended in 40 mL of pre-cooled buffer (50 mM Tris, pH 8.0, 150 mM NaCl) and disrupted by sonication for 30 min, after which the inclusion bodies containing Plychap001 were collected by centrifugation at 16,000× *g* and 4 °C for 10 min. The inclusion bodies were dissolved using high concentration urea buffer (8 M urea, 50 mM Tris-HCl, pH 8.0, 150 mM NaCl) and ultrasonication for another 10 min. Affinity purification was performed with Ni^2+^ Sepharose™6 Fast Flow column (Sangon Biotech, Shanghai, China), in which washing was performed using gradient concentration of wash buffer (8 M urea, pH 8.0, 50 mM Tris-HCl, 150 mM NaCl, 10–500 mM imidazole). Afterward, the purified protein was renatured in dialysate buffer (50 mM Tris, pH 8.0, 150 mM NaCl, 2 mM EDTA, 4 mM reduced glutathione (GSH), 0.4 mM oxidized glutathione (GSSG), 0.4 M L-arginine, 10% (*v/v*) glycerol). Finally, solution containing purified Plychap001 was dialyzed against storage buffer (50 mM Tris, pH 8.0, 150 mM NaCl, 20%(*v/v*) glycerol) and stored at −20 °C. The concentration of recombinant Plychap001 was determined using Bradford Protein Assay Kit (Solarbio Co., Beijing, China).

### 2.3. Identification of Recombinant Plychap001

Sodium dodecyl sulfate-polyacrylamide gel electrophoresis (SDS-PAGE) was used to separate and identify the recombinant Plychap001. After running SDS-PAGE gels, protein bands were stained using Coomassie Brilliant Blue R-250 (Sigma, St Louis, MO, USA) to verify the target protein bands in the elution. Afterwards, western blotting was performed following the previously described procedure [25] to confirm the presence of recombinant Plychap001. Plychap001 bands from the unstained gels were transferred onto PVDF membranes, after which the primary rabbit anti-His IgG antibody (1:5000 dilution) and the secondary goat anti-rabbit antibody (1:8000 dilution) were successively incubated with the membranes. Pictures were taken after the addition of Pierce™ ECL Western Blotting Substrate (Thermo Fisher Scientific, Carlsbad, MA, USA) to the membranes. In addition, the target protein band was used for peptide identification using high-performance liquid chromatography coupled with the Q ExactiveTM Plus (Thermo Fisher Scientific, Carlsbad, MA, USA). Proteome Discoverer 1.3 and MaxQuant v1.6.5.0 were used to analyze the multiple peaks against the phage qdvp001 proteins database in NCBI (https://www.ncbi.nlm.nih.gov/protein/?term=Vibrio+phage+qdvp001, NCBI database as of October 2020), all peptides originating from Plychap001 identified by LC-MS/MS were listed in Table 1.

### 2.4. Anti-V. parahaemolyticus Activity Assay

To test anti-*V. parahaemolyticus* activity of Plychap001, *V. parahaemolyticus* 17802 was cultured until OD_600 nm_ reached 0.8. Bacterial cells were subsequently centrifuged at 4000× *g* for 5 min, washed thrice and resuspended in PBS at the final optical density OD_600 nm_ of 1.0 (approximately 8 × 10^8^ CFU/mL). Afterwards, 100 μL of *V. parahaemolyticus* cultures were added to 90 μL of PBS. Aliquots (10 μL) of Plychap001 at different concentrations were added to the bacterial solutions. These mixtures of bacterial solutions and Plychap001 were then ten-fold diluted and coated onto Zobell 2216 marine agar (Hopebio, Qingdao, China) plates after 15 min-incubation at 37 °C. Single colonies on the plates were counted for the residual viable cell numbers (CFUs) the next day. Same volumes of dilution buffer were used as negative controls. A decrease in visible bacterial counts was used to calculate the antibacterial activity. All experiments were performed in triplicate.

### 2.5. Stability of Recombinant Plychap001

To test the stability of Plychap001 under different pH conditions, 10 μL of 1 mg/mL Plychap001 was added into 90 μL buffers under serial pH conditions (50 mM sodium acetate for pH 4.0–6.0 and 50 mM Tris-HCl for pH 7.0–11.0) and then incubated at 37 °C for 15 min. The effect of different concentrations of EDTA in the dilution buffer (50 mM Tris-HCl, 150 mM NaCl, pH 8.0, 0–5 mM EDTA) on the lytic activity of Plychap001 was also tested. To test the effect of temperature on the stability of Plychap001, 20 μL of 1 mg/mL Plychap001 was incubated at different temperatures (4–80 °C) for 15 min. After normalization to room temperature, the Plychap001 was mixed with 180 μL of *V. parahaemolyticus* cultures to assess the antibacterial activity of Plychap001. For saline concentrations challenge, 10 μL of 1 mg/mL Plychap001 was added into 90 μL of *V. parahaemolyticus* cultures containing different NaCl concentrations (50 mM Tris-HCl, pH 8.0, 0–500 mM NaCl) at 37 °C for 15 min. Anti-*V. parahaemolyticus* activity assay was the same as previously described (M&M, Section 2.4) and the relative antibacterial activity was calculated by dividing the activity of a test group with the control group (100% activity). All experiments were performed in triplicate.

### 2.6. Turbidity Reduction Assay

*V. parahaemolyticus* 17802 was cultured until OD_450 nm_ reached 0.8. Bacterial cells were centrifuged at 4000× *g* for 5 min, washed thrice, and resuspended in PBS to adjust OD_450 nm_ to approximately 1.1. Cell suspension of 100 μL was mixed with 100 μL of Plychap001 stock (0.2 mg/mL) in a sterile polystyrene 96-well micro-assay plate. The activity tests of lysozyme (0.2 mg/mL) and the dialysate buffer (M&M, Section 2.2) were used as controls. Bacterial cultures pretreated with EDTA before centrifugation were used to reduce the barrier effect of the outer membrane of *V. parahaemolyticus*. All samples were incubated at 37 °C with shaking and the reduction in the optical density at 450 nm was measured using a multi-mode reader (Synergy LX, BioTek Instruments, Inc., Winooski, VT, USA).

### 2.7. Anti-Microbial Spectrum of Plychap001

Twenty reference strains used to test the anti-microbial spectrum are listed in Table 2. To test the bactericidal effect of the qdvp001 phage and Plychap001, 3 μL of the qdvp001 phage (8 × 10^8^ PFU/mL) and 3 μL of Plychap001 solution (0.1 mg/mL) were spotted onto a freshly prepared bacterial lawn on Zobell 2216 marine agar (Hopebio, Qingdao, China) plates, respectively. After incubating the plates at 25 °C for 24 h, the qdvp001 phage and Plychap001 were determined to be capable of killing the tested bacterial strain if a clear inhibition zone was observed on the bacterial lawn.

### 2.8. Analysis of the Bactericidal Effects of Plychap001 on V. parahaemolyticus Biofilm

Crystal violet staining assay was used to assess the bactericidal effects of Plychap001 on *V. parahaemolyticus* biofilm. Aliquots (1 μL) of *V. parahaemolyticus* 17802 cell suspensions (10^8^ CFU/mL) were mixed with 199 μL TSB media with 3% NaCl in 96-well micro-assay plate and incubated at 37 °C without shaking for 72 h to form mature biofilms. The media was gently removed and the wells were washed three times with PBS. Different concentrations of Plychap001 were then added to the micro-assay plate followed by another incubation at 37 °C for 2 h. The wells were afterwards gently washed thrice with 200 μL sterile 0.9% normal saline. After dehydrating at 60 °C for 30 min, aliquots (200 μL) of 0.1% (*w/v*) crystal violet were added to each well, followed by incubation at room temperature for 30 min. The excess stain was washed with sterile distilled water and dried. Subsequently, 200 μL of 33% (*v/v*) glacial acetic acid was added and left for 15 min at room temperature to release the stain from adherent cells into each well. The amount of biofilm was described using the value of absorbance at OD_595 nm_ measured by a multi-mode reader (Synergy LX, BioTek Instruments, Inc., Winooski, VT, USA).

### 2.9. Scanning Electron Microscope (SEM) Observation of Plychap001-Treated V. parahaemolyticus Biofilm

Biofilm samples for SEM observation were prepared based on the previous procedure [26] with some modifications. The sterilized glass coupons were used to provide an effective place for the adhesion of *V. parahaemolyticus* biofilm. After various treatments, the glass coupons with mature biofilms were gently taken out and washed thrice with sterile 0.9% saline distilled water, and then transferred onto a new 24-well micro-assay plate. Each well was treated with or without Plychap001 (0.1 mg/mL) at 37 °C for 2 h, then gently washed thrice with sterile 0.9% saline distilled water. Afterward, biofilms on glass coupons were fixed with 3% glutaraldehyde for 4 h, washed gently with PBS, and fixed with 1% osmic acid for another 1.5 h. Next, biofilms were further washed, dried, and then coated with gold to complete the preparation of observation samples. SEM characterization was performed on TESCAN VEGA3 (TESCAN, Brno, the Czech Republic).

### 2.10. Statistical Analysis

All experiments were performed in triplicate. Obtained data were expressed as mean ± standard deviation. The results were analyzed with the one-way analysis of variance (ANOVA) and Tukey’s range test. A *p*-value of < 0.01 was considered statistically significant. All statistical analyses were performed using GraphPad Prism 8.0 software (GraphPad, San Diego, CA, USA).

## 3. Results

### 3.1. Modular Structure of Endolysin Lysqdvp001

**Figure 1:**
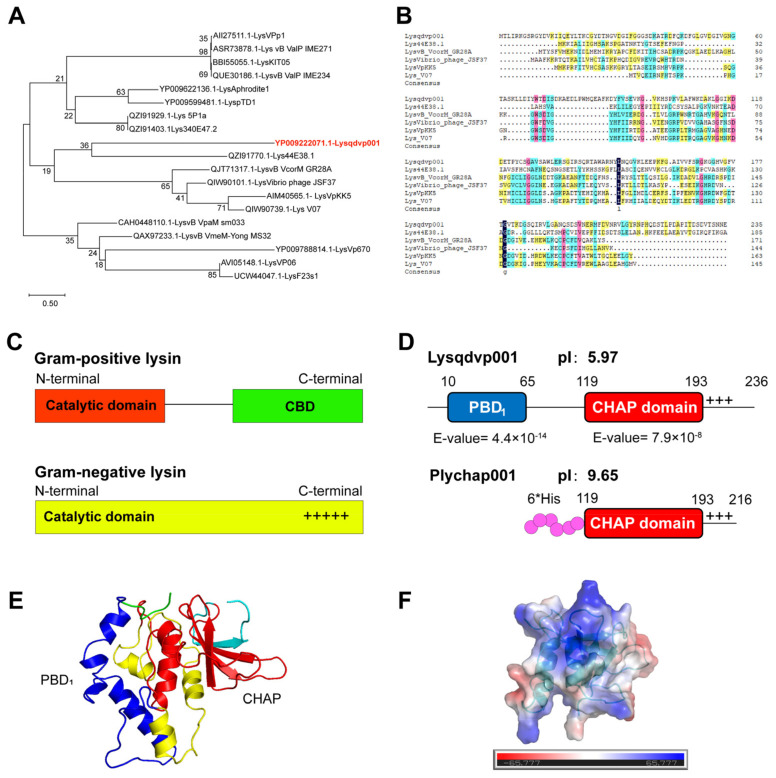
Bioinformatic analysis of Lysqdvp001. (**A**) Phylogenetic tree showing the evolutionary relationship of Lysqdvp001 and related *V. parahaemolyticus* lysins. Amino acid sequences were aligned using Clustal W and the phylogenetic relationships were calculated by the neighbor-joining method using MEGA 11 software. (**B**) Amino acid sequence alignment of endolysins in Clustal W. The sequences of Lysqdvp001 and related endolysins were retrieved from GenBank. (**C**) Schematic illustration of typical Gram-positive and Gram-negative bacterial endolysins. (**D**) Schematic illustration of Lysqdvp001 and Plychap001. The domain arrangement of Lysqdvp001 was predicted by Pfam analysis to contain a peptidoglycan-binding domain (PBD_1_, amino acid residues 10–65) and a CHAP domain (amino acid residues 119–193). Recombinant Plychap001 construct contains a 6 × His-tag and amino acid residues 119–216 of Lysqdvp001. (**E**) 3D-structural model of Lysqdvp001 predicted in SWISS-MODEL and modified in PyMOL. The N-terminal PBD1 domain and the C-terminal catalytic CHAP domain are colored blue and red, respectively. The protein segment interconnecting these two domains is colored yellow and the N-terminal and C-terminal tails of Lysqdvp001 are colored green and sky blue, respectively. (**F**) 3D-structural model of Plychap001 predicted in SWISS-MODEL and modified in PyMOL. Electrostatic surface charge is shown as blue (positive charge), red (negative charge) and white (neutral charge).

The phylogenetic relationship between Lysqdvp001 and other related *V. parahaemolyticus* lysins was analyzed using Clustal W and indicated low similarity to other compared lysins (Figure 1A). Multiple sequence alignment (Figure 1B) of Lysqdvp001 and most closely related phage lysins further demonstrated that the conserved amino acid residues IIe149 and Gly179, which located in the CHAP domain, may play important roles in the lytic activity of Lysqdvp001. Gram-positive lysins are usually encoded with modular structure composing a cell wall binding domain (CBD) and one or two catalytic domains (Figure 1C) [15,18]. Conversely, Gram-negative lysins usually consist of an independent catalytic domain with C-terminal charged residues responsible for binding to bacterial cell wall (Figure 1C) [16,17,24]. Pfam analysis indicated that Lysqdvp001 contains two conserved domains, namely an N-terminal putative peptidoglycan (PG)-binding domain (pfam01471, 52.4 bits, e-value: 4.4 × 10^−14^) and a C-terminal CHAP domain (cysteine, histidine-dependent amidohydrolases/peptidases, cd00254, 40.5 bits, e-value: 7.9 × 10^−8^) (Figure 1D). Thus, Lysqdvp001 also appears to possess modular structure similar with Gram-positive bacterial endolysins, albeit its domains are arranged in opposite order. 3D structural modeling of Lysqdvp001 using SWISS-MODEL suggested that both domains of Lysqdvp001 may adopt typical α-helical fold (Figure 1E). The construct tested for bactericidal activity against *V.parahaemolyticus*, henceforth referred to as Plychap001, includes CHAP domain and part of the C-terminal sequence of Lysqdvp001 and an N-terminal 6 × His-tag at the N-terminal (Figure 1D) and its predicted molecular weight is 11.8 kDa. Analysis of surface charge distribution on the predicted structural model of Plychap001 suggested that its C-terminus is positively charged (Figure 1F).

### 3.2. Expression, Purification and Identification of Recombinant Plychap001

**Figure 2:**
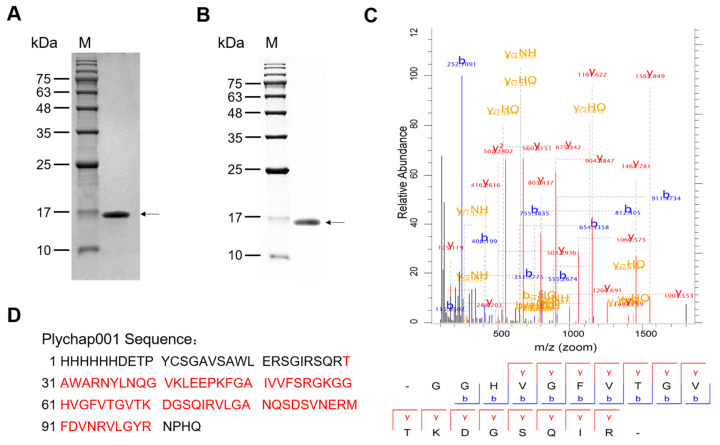
Expression and identification of Plychap001. (**A**) SDS-PAGE gel (12%) analysis of recombinant Plychap001 after purification and renaturation. (**B**) Detection of recombinant Plychap001 using western blotting. (**C**) Fragment ion spectrum of the GGHVGFVTGVTKDGSQIR peptide, i.e., one of the peptides matched with Plychap001. (**D**) Peptide fragments corresponding to Plychap001 amino acid sequence (indicated in red) identified in mass spectrometry. M: marker, indicated in the left margin in kDa.

Recombinant Plychap001 was overexpressed from pET-30a plasmid in *E. coli* BL21 (DE3) cells. The target protein exhibited high expression levels but aggregated into inclusion bodies. After denaturation, purification with nickel affinity chromatography and renaturation, a clear protein band of approximately 16 kDa, which was larger than the predicted molecular size (11.8 kDa), could be observed on SDS-PAGE gel with high purity (>90%) (Figure 2A) and a final yield of 3 mg/L. Immunoblotting was then performed to detect the presence of a 6 × His-tag at the N-terminus of the purified protein using rabbit anti-His IgG antibody. A single band at ~16 kDa was detected (Figure 2B), which is consistent with SDS-PAGE results. Afterwards, the identity of purified protein was further verified with LC-MS/MS by extracting and digesting target protein bands from the gel (Figure 2A). The detected peptides were then matched against the NCBI database; a total of 14 different peptides were identified with 68.27% amino acid sequence coverage (Figure 2D). As is shown in Figure 2C one of the typical peptides detected by mass spectrometry (GGHVGFVTGVTKDGSQIR) was mapped to residues 59–76 of Plychap001. These results indicate that recombinant Plychap001 was successfully expressed and purified.

### 3.3. Antibacterial Activity of Recombinant Plychap001

**Figure 3:**
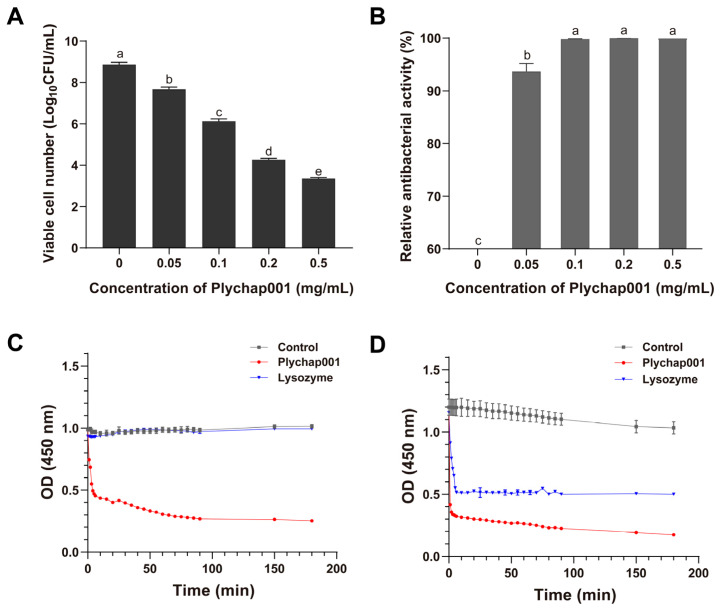
Antibacterial activity of recombinant Plychap001. (**A**) Statistical results of viable cell number after treatment with different concentrations of Plychap001. (**B**) Relative antibacterial activity was calculated as percentage by dividing the residual viable cells number with total cell number. (**C**,**D**) Results of turbidity reduction assays. (**C**) OD_450 nm_ of *V. parahmolyticus* suspensions treated with Lysqdvp001 or Lysozyme. (**D**) OD_450 nm_ of *V. parahmolyticus* suspensions pretrated with EDTA and treated with Lysqdvp001 or Lysozyme. Different letters (a–e) on the columns indicated significant difference between each other at *p* < 0.01 level. Data were shown as the mean ± SEM for n = 3.

To assess the direct antibacterial activity of purified Plychap001, more than 8 log units of *V. parahaemolyticus* 17802 cells were treated with different concentrations of Plychap001 without pretreatment with any OMPs. Antibacterial activity was expressed as the decrease of viable cell numbers. As depicted in Figure 3A, the viable bacterial count substantially decreased proportionally to the increase in Plychap001 concentration. Plychap001 (0.5 mg/mL) could bring about more than 5 log reduction of exponentially growing cells in vitro within 15 min, indicating efficient antibacterial activity against *V. parahaemolyticus* 17802. Within the same time frame, 0.1 mg/mL of Plychap001 was found to achieve a 2 log reduction of *V. parahaemolyticus*, which approximates to 100% sterilization (Figure 3B) and was thus chosen as the working concentration in subsequent experiments. The lytic activity of Plychap001 against *V. parahaemolyticus* 17802 cells was further tested in turbidity reduction assay. At 37 °C (Figure 3C), no significant decrease in OD_450 nm_ values was observed in control and 0.1 mg/mL Lysozyme-treated samples during 0 to 3 h. The OD_450 nm_ values of bacterial samples treated with 0.1 mg/mL Plychap001 dropped from about 0.99 to 0.25 during the same treatment period. To remove the barrier effect of the outer membrane, we repeated the turbidity assay with *V. parahaemolyticus* cells pretreated with EDTA. Initial OD_450 nm_ values of the bacterial suspensions were close to 1.10. After 3 h of treatment, the OD_450 nm_ values of the control group and samples treated with 0.1 mg/mL Lysozyme and 0.1 mg/mL Plychap001 were 1.03, 0.50, and 0.17, respectively (Figure 3D). Together, these findings suggest that Plychap001 can efficiently kill *V. parahaemolyticus* 17802 cells by carrying out cell lysis.

### 3.4. Stability of Recombinant Plychap001

**Figure 4:**
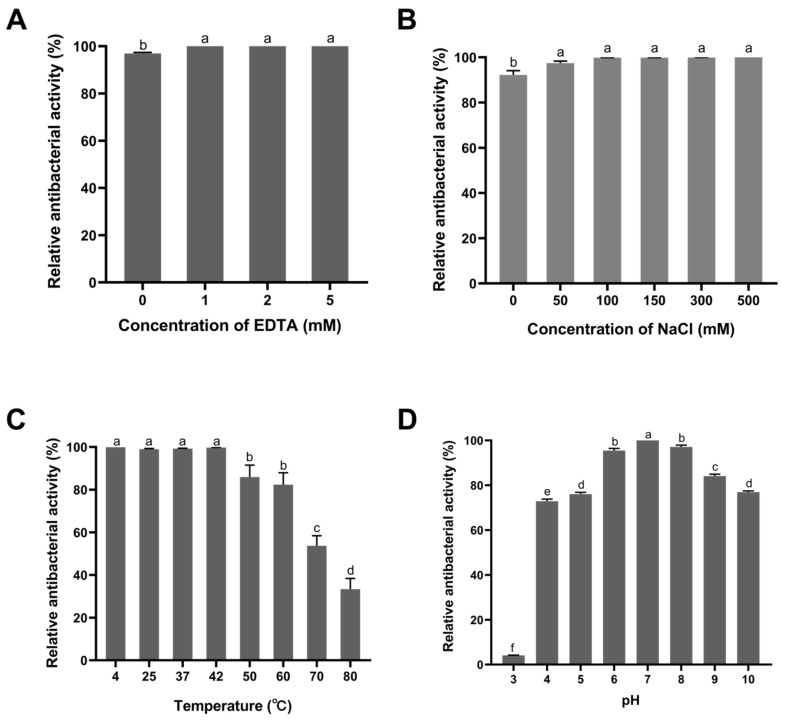
Effects of key parameters on the lytic activity of recombinant Plychap001. Lytic activity of Plychap001 (0.1 mg/mL) against *V*. *Parahaemolyticus* 17802 in varying (**A**) concentrations of EDTA, (**B**) concentrations of NaCl, (**C**) temperature, and (**D**) pH value. Different letters (a–f) on the columns indicate significant difference between each other at *p* < 0.01 level. Data were shown as the mean ± SEM for n = 3.

Stability and antibacterial activity of recombinant Plychap001 was tested by carrying several key reaction parameters, including (1) concentration of EDTA, (2) concentration of NaCl in the reaction solution, (3) temperatures, and (4) pH value of the reaction solution. Relative antibacterial activity was calculated by dividing viable cell number by the total cell numbers. Upon treatment with 1 mM or higher concentrations of EDTA (Figure 4A), nearly 100% of high-density *V. parahaemolyticus* 17802 cells were killed by Plychap001, a value significantly higher than that of the control, suggesting that the addition of EDTA could further enhance the antibacterial activity of Plychap001. NaCl was selected to test saline concentration effect, the results revealed that different NaCl concentrations (0 to 500 mM) did not have a significant impact on the bactericidal activity of Plychap001 (Figure 4B); recombinant Plychap001 exhibited approximately 100% lytic activity even under 500 mM NaCl. The thermal stability of recombinant Plychap001 was determined by incubation with *V. parahaemolyticus* 17802 cells at temperatures for 15 min. The results demonstrated that Plychap001 displays approximately 100% activity within the 4–42 °C range and more than 80% relative activity at 60 °C (Figure 4C). The activity of recombinant Plychap001 sharply decreased as the temperatures reached 70 °C and above, but Plychap001 was still able to kill approximately 30% of *V. parahaemolyticus* cells when heating the reaction mixture at 80 °C for 15 min. As is shown in Figure 4D, Plychap001 exhibited high antibacterial activity between pH 4.0 and 10.0, with the highest activity detected at pH 7.0. The activity of Plychap001 significantly decreased to less than 10% at pH value of 3.0.

### 3.5. Antibacterial Spectrum of Plychap001

In order to investigate the antibacterial spectrum of Plychap001, recombinant Plychap001 was tested against twenty strains of Gram-negative bacteria (including eleven *V. parahaemolyticus* strains) and one species of Gram-positive bacteria (*Staphylococcus aureus*) (Table 2). The results demonstrated that Plychap001 could kill not only all tested *V. parahaemolyticus* strains but also several other species of Gram-negative bacteria, including *Shewanella baltica*, *Vibrio harveyi*, and *Acinetobacter baumannii*. In comparison, the qdvp001 phage could only successfully lyse against three out of eleven tested *V. parahaemolyticus* strains. Thus, the antibacterial spectrum of Plychap001 is significantly broader than that of the qdvp001 phage.

### 3.6. Plychap001 Can Effectively Degrade Mature V. parahaemolyticus Biofilm

**Figure 5:**
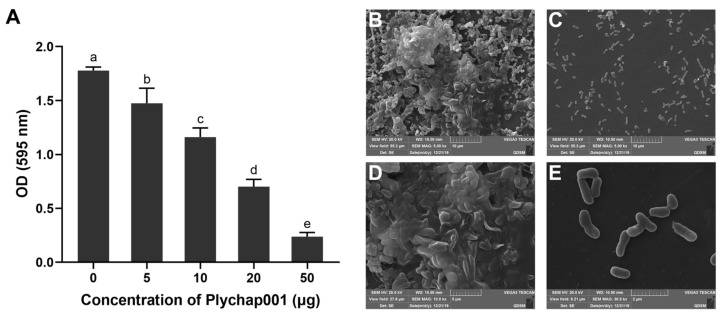
Antibacterial effect of Plychap001 on a mature *V. parahaemolyticus* 17802 biofilm. (**A**) Biofilm mass was determined using plating and crystal-violet staining methods. (**B**,**D**) SEM images of untreated *V. parahaemolyticus* biofilm. (**C**,**E**) SEM images of *V. parahaemolyticus* biofilm after treatment with 0.1 mg/mL Plychap001. Different letters (a–e) on the columns indicated significant difference between each other at *p* < 0.01 level. Data were shown as the mean ± SEM for *n* = 3.

To test the antibacterial effects of recombinant Plychap001 on *V. parahaemolyticus* biofilm, the bacterial culture was attached to microplate wells to facilitate biofilm formation. After 72 h of standing culture, different concentrations of Plychap001 were added to biofilm samples, followed by incubation for additional 2 h. The efficiency of biofilm degradation was dose-dependent (Figure 5A). Initial biofilm mass (determined by crystal violet staining) was up to 1.78, but the optical density (OD_595 nm_) staining of the biofilm was reduced significantly upon the addition of Plychap001, reaching 86.61% upon the addition of 50 μg (0.25 mg/mL) of Plychap001. SEM imaging further confirmed the formation of *V. parahaemolyticus* biofilm tightly attached to polystyrene surface (Figure 5B,D).Furthermore, SEM images taken 2 h of Plychap001 treatment indicate that the biofilm was almost complete eliminated, leaving only a few bacterial cells (Figure 5C,E).

## 4. Discussion

*V. parahaemolyticus* is one of the major pathogens causing food poisoning all over the world [1,2,3]. Multi-drug resistant *V. parahaemolyticus* has become a big threat to public health and global economics, especially to the food industry [27]. Long-term co-evolution of phages and host bacteria has allowed endolysins to develop efficient bacterial cell wall cleavage ability and lower host tolerance [14]. Therefore, endolysins are considered a promising strategy for tackling the antibiotic resistance problem. However, the outer membranes of Gram-negative bacteria still represent a great obstacle that hinders the extracellular application of endolysins against Gram-negative bacteria [19,20,21]. Here, a single recombinant CHAP domain of Lysqdvp001, named Plychap001, purified, identified, and tested against *V. parahaemolyticus* and its biofilm.

Firstly, the bioinformatic analysis and recombinant expression of Plychap001 were performed. Lysqdvp001 has been reported as a highly efficient endolysin against various *V. parahaemolyticus* strains [28]. However, without pretreatment with OMPs, the extracellular application of Lysqdvp001 alone could not inhibit the growth of *V. parahaemolyticus* due to the presence of physical barrier formed by the outer membrane [21]. Genome analysis of the qdvp001 phage revealed the absence gene sequences encoding holin or other proteins that assist with the lytic function, indicating that Lysqdvp001 lyses PG directly and independently during phage infection. Independent and direct lytic activity has also been reported for some other endolysins, including LysPA26 against *P. aeruginosa* [24] and LysSTG2 against *S. typhimurium* [17]. The phylogenetic tree showed a low similarity of Lysqdvp001 with other related *V. parahaemolyticus* lysins (Figure 1A), suggesting distinct structural and functional characteristics of Lysqdvp001. Accordingly, Lysqdvp001 was predicted to have a modular structure consisting of an N-terminal binding domain and a C-terminal catalytic domain (Figure 1D). To the best of our knowledge, Lysqdvp001 is the first reported endolysin targeting *V. parahaemolyticus* with modular structure [28] that may be somewhat similar to the structures of *Staphylococcus aureus*-targeting LysGH15 [15] and *Enterococcus faecalis*-targeting [18]. Moreover, the single CHAP domains of LysGH15 and LysIME-EF1 have shown an independent effect on the cleavage of host cells. The predicted 3D-structural model of Lysqdvp001 suggested a typical α-helical fold of the CHAP domain (Figure 1E,F), which has been previously reported to be positively charged amphipathic and responsible for the interaction with the outer membrane [16,29]. Thus, we hypothesized that the single CHAP domain of Lysqdvp001 may carry out direct lysis against *V. parahaemolyticus* cells without the need for pretreatment with OMPs. To verify this conjecture, we expressed recombinant Plychap001 in *E. coli* and obtained it at high purity (>90%) (Figure 2A). The identity of target protein band was further identified and confirmed by western blotting (Figure 2B) and mass spectrometry (Figure 2C).

**Scheme 1:**
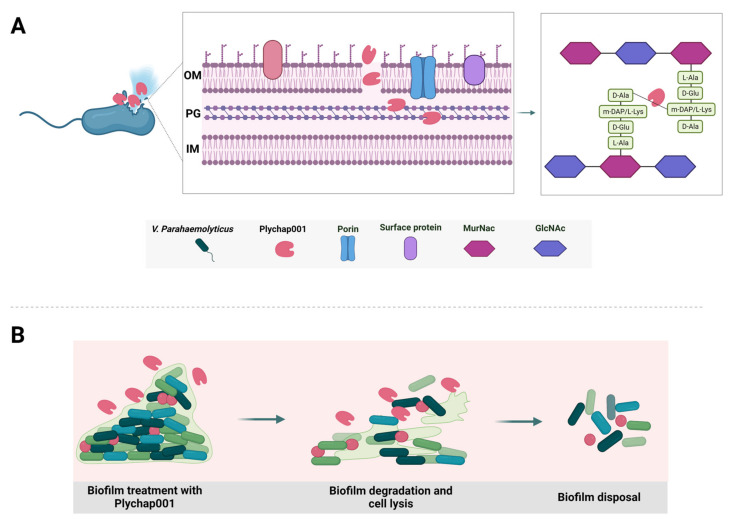
(**A**) Schematic diagram depicting the extracellular application of Plychap001 direct lysis of *V. parahaemolyticus* cell structure. (**B**) Schematic diagram illustrating degradation of mature *V. parahaemolyticus* mature using Plychap001. Both diagrams were generated on BioRender.com, April 2022.

Secondly, the anti-*V. parahaemolyticus* activity of recombinant Plychap001 was evaluated. As was shown in Figure 3A, the number of *V. parahaemolyticus* 17802 cells in exponential growth phase decreased by more than 2-log-unit upon 15-min treatment with 0.1 mg/mL or higher concentrations of Plychap001. The working concentration of Plychap001 at 0.1 mg/mL (around 6.25 μM) (Figure 3B), is lower than another single domain endolysin-LysPA26 (0.5 mg/mL) [24], but displayed a higher lytic activity. Lytic effect of Plychap001was further tested using turbidity reduction assay (Figure 3C), the OD_450 nm_ of samples treated with 0.1 mg/mL Plychap001 showed a significant decrease from 0.99 to 0.25 within 3 h. These results indicated a direct and high lytic activity against *V. parahaemolyticus* without pretreatment with any OMPs. While the ability to kill bacteria without the use of OMPs was also reported for several other endolysins, including LysPA26, LysSTG2, and ABgp46 [17,24,29], the mechanisms underlying the bactericidal activity of these endolysins remain unclear. Several explanations have been proposed for this phenomenon: (1) endolysins with a C-terminal amphipathic α-helix may disrupt outer membrane [16]; (2) the C-terminal positively charged region of the endolysins could enable the N-terminal enzymatic domain to pass through the outer membrane [29]; (3) the N-terminal extension is critical for Ts2631 endolysin, which allows the enzyme to pass through the outer membrane and interact with murein [30,31]; (4) some endolysins could use their N-terminal extension to form nanopores in the cytoplasmic membrane, killing bacteria by destabilizing the cell membrane through electrostatic interactions [32]. Taken into account, the results of bioinformatics analysis of Plychap001, a possible explanation is that Plychap001 would be positively charged in a pH 8.0 buffer due to its high PI value (9.65). Electrostatic interactions with the negatively charged outer membrane could affect membrane permeability, allowing the catalytic CHAP domain to access peptidoglycan and lyse it (Scheme 1A).

Next, the stability of recombinant Plychap001 was evaluated by various tests. When simultaneously treated with EDTA, the tested cells were more sensitive to Plychap001 (Figure 4A), indicating that EDTA could enhance the efficiency of Plychap001, which is consistent with Lysqdvp001 [21] and some other Gram-negative endolysins [33,34]. Moreover, the lytic effect of Plychap001 was not inhibited when reaction solutions contained different concentrations of NaCl (even up to 500 mM) (Figure 4B), which is even more applicative due to the marine halophilic characteristics of *V. parahaemolyticus* [35]. Temperature tests showed that Plychap001 could maintain high antibacterial activity between 4 °C and 42 °C (Figure 4C). The lysis efficiency began to decline with the increase of temperature, but Plychap001 could still kill more than 30% *V. parahaemolyticus* cells when tested at 80 °C for 15 min, meaning that Plychap001 has good stability within wide temperature range. Concurrently, the efficient cleavage ability at low temperature could contribute to the application of Plychap001 in food preservation against *V. parahaemolyticus*. The pH tests revealed that Plychap001 was highly stable at a wide pH range (4.0–10.0) (Figure 4D) and that the optimal pH for lytic activity was around 7.0. These results demonstrated that Plychap001 was highly stable under different reaction conditions, which is consistent with some other alkalophilic endolysins [19,24,36]. These characteristics also ensure Plychap001 is a promising candidate for combating *V. parahaemolyticus*.

Furthermore, the antibacterial spectrum of recombinant Plychap001 was determined. Phage specificity leads to a narrow host range which limits its application to some extent. However, the cleavage range of endolysin is usually broader than that of the parent phage [37]. Plychap001 showed the bactericidal effect not only against all tested *V. parahaemolyticus* strains (11/11), but also some other species of Gram-negative bacteria, such as *Shewanella baltica*, *Vibrio harveyi,* and *Acinetobacter baumannii*. The parent phage qdvp001 revealed a narrower host range of three out of eleven tested strains, suggesting that Plychap001 has broader antibacterial spectrum (Table 2). The antibacterial ability of Plychap001 thus has certain degree of universality among Gram-negative bacteria, which also makes it a promising alternative for the control of specific bacterial species and strains. 

Finally, the potential effect of Plychap001 on mature biofilm eradication was investigated. *V. parahaemolyticus* is known as one of the Gram-negative bacteria with the ability to form biofilms, which highly strengthens its resistance and persistence in different environments such as in food industry. Endolysins are considered as attractive agents for the prevention and eradication of bacterial biofilm [13,24,36]. In this study, Plychap001 displayed a high efficiency on the mature biofilm of *V. parahaemolyticus* 17802—86.61% of *V. parahaemolyticus* mature biofilms on polystyrene surface was removed after treatment with 0.5 mg/mL Plychap001 for 2 h (Figure 5A). The results revealed a stronger biofilm reduction capacity of Plychap001 than that of the combined treatment with Lysqdvp001 and ε-PL (55.13%) [38]. Moreover, SEM images revealed that only a few *V. parahaemolyticus* cells were left after Plychap001 treatment (Figure 5C,E). These results revealed that Plychap001 can efficiently kill *V. parahaemolyticus* cells in mature biofilms and degrade this kind of biofilms at a certain concentration (Scheme 1B).

## 5. Conclusions

Endolysins are regarded as promising agents for combating multidrug-resistant bacteria. However, the outer membrane represents a physical barrier that still greatly inhibits the application of endolysins against Gram-negative bacteria. In this study, single catalytic domain construct Plychap001 derived from the modular Gram-negative bacteria-targeting endolysin Lysqdvp001 was successfully expressed and purified. Recombinant Plychap001 exhibited efficient lytic activity against multiple strains of *V. parahaemolyticus* in vitro and was also able to efficiently degrade mature *V. parahaemolyticus* biofilm. Plychap001 was also found to maintain stability and high activity at wide temperature and pH ranges and high concentrations of NaCl. Although further investigation is required to clarify the molecular mechanism underlying the lytic activity of Plychap001, the findings of this study demonstrated that Plychap001 is an attractive strategy for controlling the multidrug-resistant *V. parahaemolyticus* in the food industry.

## Figures and Tables

**Table 1:** List of peptides originating from Plychap001 that were identified by LC-MS/MS.

Annotated Sequence	Protein	Mass	PSMs	Confidence	Score
GGHVGFVTGVTKDGSQIR	1	1814.95	19	High	5.50
GKGGHVGFVTGVTK	1	1343.74	24	High	4.50
DGSQIRVLGANQSDSVNERMFDVNR	1	2807.35	1	High	4.47
VLGANQSDSVNER	1	1388.68	187	High	4.04
GGHVGFVTGVTK	1	1158.63	294	High	3.82
NYLNQGVKLEEPK	1	1531.81	7	High	3.58
VLGANQSDSVNER	1	1389.66	5	High	3.23
FGAIVVFSR	1	995.57	109	High	2.94
NYLNQGVKLEEPK	1	1532.80	9	High	2.68
DGSQIRVLGANQSDSVNER	1	2045.00	2	High	2.64
VLGANQSDSVNERMFDVNRVLGYR	1	2739.36	3	High	2.51
VLGANQSDSVNERMFDVNR	1	2151.02	1	High	2.42
NYLNQGVK	1	935.49	8	High	2.00
TAWARNYLNQGVK	1	1520.80	2	High	1.04

PSMs, number of secondary spectra.

**Table 2:** Antibacterial spectra of Lysqdvp001 phage and recombinant Plychap001.

Bacterial Isolate	Activity		Source ^b^
	qdvp001 Phage ^a^	Plychap001 ^a^	
*VP* 1.1	−	+	isolated from sewage
*VP* 1.3	−	+	isolated from sewage
*VP* 2.2	−	+	isolated from sewage
*VP* 3.1	−	+	isolated from sewage
*VP* 3.2	−	+	isolated from sewage
*VP* 4.1	−	+	isolated from sewage
*VP* 304	+	+	stored in the lab
*VP* 461	+	+	stored in the lab
*VP* 800	−	+	stored in the lab
*VP* l4-90	−	+	stored in the lab
*V. parahaemolyticus* 17802	+	+	ATCC
*Vibrio harveyi* V1	−	+	stored in the lab
*Shewanella baltica* S1	−	+	stored in the lab
*Acinetobacter baumannii* A1	−	+	stored in the lab
*Staphylococcus aureus* 43300	−	−	ATCC
*Pseudomonas fluorescens* P1	−	−	stored in the lab
*Aeromonas hydrophila* A1	−	−	stored in the lab
*Pseudomonas putrefaciens* P1	−	−	stored in the lab
*Salmonella typhimurium* 14028	−	−	ATCC
*Vibrio anguillarum* V1	−	−	stored in the lab

^a^ +, bacteriostatic ring observed; −, no bacteriostatic ring observed. ^b^ ATCC, American Type Culture Collection.

## Data Availability

Data is contained within the article.

## References

[1] Banu S.F., Rubini D., Murugan R., Vadivei V., Gowrishankar S., Pandian S.K., Nithyanand P. (2018). Exploring the antivirulent and sea food preservation efficacy of essential oil combined with DNase on Vibrio parahaemolyticus. LWT Food Sci. Technol..

[2] Elmahdi S., DaSilva L.V., Parveen S. (2016). Antibiotic resistance of Vibrio parahaemolyticus and Vibrio vulnificus in various countries: A review. Food Microbiol..

[3] Lei T., Jiang F., He M., Zhang J., Zeng H., Chen M., Pang R., Wu S., Wei L., Wang J. (2020). Prevalence, virulence, antimicrobial resistance, and molecular characterization of fluoroquinolone resistance of Vibrio parahaemolyticus from different types of food samples in China. Int. J. Food Microbiol..

[4] Li R., Lin D., Chen K., Wong M.H., Chen S. (2015). First detection of AmpC beta-lactamase bla(CMY-2) on a conjugative IncA/C plasmid in a *Vibrio parahaemolyticus* isolate of food origin. Antimicrob. Agents Chemother..

[5] Paterson D.L., Harris P.N.A. (2016). Colistin resistance: A major breach in our last line of defence. Lancet Infect. Dis..

[6] van Hal S.J., Willems R.J.L., Gouliouris T., Ballard S.A., Coque T.M., Hammerum A.M., Hegstad K., Westh H.T., Howden B.P., Malhotra-Kumar S. (2021). The global dissemination of hospital clones of *Enterococcus faecium*. Genome Med..

[7] Lei T., Zhang J., Jiang F., He M., Zeng H., Chen M., Pang R., Wu H., Wu S., Wang J. (2020). Characterization of class 1 integrons harboring blaVEB-1 in *Vibrio parahaemolyticus* isolated from ready-to-eat foods in China. Int. J. Food Microbiol..

[8] Shangguan W., Xie T., Zhang R., Lu C., Han X., Zhong Q. (2021). Anti-biofilm potential of kefir-derived Lactobacillus paracasei L10 against *Vibrio parahaemolyticus*. Lett. Appl. Microbiol..

[9] Rahman M.U., Wang W., Sun Q., Shah J.A., Li C., Sun Y., Li Y., Zhang B., Chen W., Wang S. (2021). Endolysin, a Promising Solution against Antimicrobial Resistance. Antibiotics.

[10] Shannon R., Radford D.R., Balamurugan S. (2020). Impacts of food matrix on bacteriophage and endolysin antimicrobial efficacy and performance. Crit. Rev. Food Sci. Nutr..

[11] Trudil D. (2015). Phage lytic enzymes: A history. Virol. Sin..

[12] Fursov M.V., Abdrakhmanova R.O., Antonova N.P., Vasina D.V., Kolchanova A.D., Bashkina O.A., Rubalsky O.V., Samotrueva M.A., Potapov V.D., Makarov V.V. (2020). Antibiofilm Activity of a Broad-Range Recombinant Endolysin LysECD7: In Vitro and In Vivo Study. Viruses.

[13] Zhang Y., Huang H.-H., Duc H.M., Masuda Y., Honjoh K.-i., Miyamoto T. (2022). Application of endolysin LysSTG2 as a potential biocontrol agent against planktonic and biofilm cells of Pseudomonas on various food and food contact surfaces. Food Control.

[14] Gondil V.S., Harjai K., Chhibber S. (2020). Endolysins as emerging alternative therapeutic agents to counter drug-resistant infections. Int. J. Antimicrob. Agents.

[15] Gu J., Feng Y., Feng X., Sun C., Lei L., Ding W., Niu F., Jiao L., Yang M., Li Y. (2014). Structural and biochemical characterization reveals LysGH15 as an unprecedented “EF-hand-like” calcium-binding phage lysin. PLoS Pathog..

[16] Lood R., Winer B.Y., Pelzek A.J., Diez-Martinez R., Thandar M., Euler C.W., Schuch R., Fischetti V.A. (2015). Novel phage lysin capable of killing the multidrug-resistant gram-negative bacterium *Acinetobacter baumannii* in a mouse bacteremia model. Antimicrob. Agents Chemother..

[17] Zhang Y., Huang H.H., Duc H.M., Masuda Y., Honjoh K.I., Miyamoto T. (2021). Endolysin LysSTG2: Characterization and application to control *Salmonella typhimurium* biofilm alone and in combination with slightly acidic hypochlorous water. Food Microbiol..

[18] Zhou B., Zhen X., Zhou H., Zhao F., Fan C., Perculija V., Tong Y., Mi Z., Ouyang S. (2020). Structural and functional insights into a novel two-component endolysin encoded by a single gene in *Enterococcus faecalis* phage. PLoS Pathog..

[19] Liu A., Wang Y., Cai X., Jiang S., Cai X., Shen L., Liu Y., Han G., Chen S., Wang J. (2019). Characterization of endolysins from bacteriophage LPST10 and evaluation of their potential for controlling *Salmonella typhimurium* on lettuce. LWT.

[20] Oliveira H., Thiagarajan V., Walmagh M., Sillankorva S., Lavigne R., Neves-Petersen M.T., Kluskens L.D., Azeredo J. (2014). A thermostable Salmonella phage endolysin, Lys68, with broad bactericidal properties against gram-negative pathogens in presence of weak acids. PLoS ONE.

[21] Wang W., Li M., Lin H., Wang J., Mao X. (2016). The *Vibrio parahaemolyticus*-infecting bacteriophage qdvp001: Genome sequence and endolysin with a modular structure. Arch. Virol..

[22] Ning H., Cong Y., Lin H., Wang J. (2021). Development of cationic peptide chimeric lysins based on phage lysin Lysqdvp001 and their antibacterial effects against *Vibrio parahaemolyticus*: A preliminary study. Int. J. Food Microbiol..

[23] Nie T., Meng F., Zhou L., Lu F., Bie X., Lu Z., Lu Y. (2021). In Silico Development of Novel Chimeric Lysins with Highly Specific Inhibition against Salmonella by Computer-Aided Design. J. Agric. Food Chem..

[24] Guo M., Feng C., Ren J., Zhuang X., Zhang Y., Zhu Y., Dong K., He P., Guo X., Qin J. (2017). A Novel Antimicrobial Endolysin, LysPA26, against *Pseudomonas aeruginosa*. Front. Microbiol..

[25] Sun L., Xu L., Huang Y., Lin H., Ahmed I., Li Z. (2019). Identification and comparison of allergenicity of native and recombinant fish major allergen parvalbumins from Japanese flounder (*Paralichthys olivaceus*). Food Funct..

[26] Yu P., Mathieu J., Yang Y., Alvarez P.J.J. (2017). Suppression of Enteric Bacteria by Bacteriophages: Importance of Phage Polyvalence in the Presence of Soil Bacteria. Environ. Sci. Technol..

[27] Lopatek M., Wieczorek K., Osek J. (2018). Antimicrobial Resistance, Virulence Factors, and Genetic Profiles of *Vibrio parahaemolyticus* from Seafood. Appl. Environ. Microbiol..

[28] Matamp N., Bhat S.G. (2019). Phage Endolysins as Potential Antimicrobials against Multidrug Resistant *Vibrio alginolyticus* and *Vibrio parahaemolyticus*: Current Status of Research and Challenges Ahead. Microorganisms.

[29] Oliveira H., Vilas Boas D., Mesnage S., Kluskens L.D., Lavigne R., Sillankorva S., Secundo F., Azeredo J. (2016). Structural and Enzymatic Characterization of ABgp46, a Novel Phage Endolysin with Broad Anti-Gram-Negative Bacterial Activity. Front. Microbiol..

[30] Plotka M., Kapusta M., Dorawa S., Kaczorowska A.K., Kaczorowski T. (2019). Ts2631 Endolysin from the Extremophilic Thermus scotoductus Bacteriophage vB_Tsc2631 as an Antimicrobial Agent against Gram-Negative Multidrug-Resistant Bacteria. Viruses.

[31] Plotka M., Sancho-Vaello E., Dorawa S., Kaczorowska A.K., Kozlowski L.P., Kaczorowski T., Zeth K. (2019). Structure and function of the Ts2631 endolysin of *Thermus scotoductus* phage vB_Tsc2631 with unique N-terminal extension used for peptidoglycan binding. Sci. Rep..

[32] Plotka M., Szadkowska M., Hakansson M., Kovacic R., Al-Karadaghi S., Walse B., Werbowy O., Kaczorowska A.K., Kaczorowski T. (2020). Molecular Characterization of a Novel Lytic Enzyme LysC from *Clostridium intestinale* URNW and Its Antibacterial Activity Mediated by Positively Charged N-Terminal Extension. Int. J. Mol. Sci..

[33] Jiang Y., Xu D., Wang L., Qu M., Li F., Tan Z., Yao L. (2021). Characterization of a broad-spectrum endolysin LysSP1 encoded by a *Salmonella bacteriophage*. Appl. Microbiol. Biotechnol..

[34] Ni P., Wang L., Deng B., Jiu S., Ma C., Zhang C., Almeida A., Wang D., Xu W., Wang S. (2021). Characterization of a Lytic Bacteriophage against *Pseudomonas syringae* pv. *actinidiae* and Its Endolysin. Viruses.

[35] Letchumanan V., Chan K.G., Lee L.H. (2014). *Vibrio parahaemolyticus*: A review on the pathogenesis, prevalence, and advance molecular identification techniques. Front. Microbiol..

[36] Cha Y., Son B., Ryu S. (2019). Effective removal of staphylococcal biofilms on various food contact surfaces by *Staphylococcus aureus* phage endolysin LysCSA13. Food Microbiol..

[37] O’Flaherty S., Coffey A., Meaney W., Fitzgerald G.F., Ross R.P. (2005). The recombinant phage lysin LysK has a broad spectrum of lytic activity against clinically relevant staphylococci, including methicillin-resistant *Staphylococcus aureus*. J. Bacteriol..

[38] Ning H.Q., Lin H., Wang J.X. (2021). Synergistic effects of endolysin Lysqdvp001 and epsilon-poly-lysine in controlling *Vibrio parahaemolyticus* and its biofilms. Int. J. Food Microbiol..

